# Multifunctional Nanoplatform Based on Sunitinib for Synergistic Phototherapy and Molecular Targeted Therapy of Hepatocellular Carcinoma

**DOI:** 10.3390/mi14030613

**Published:** 2023-03-07

**Authors:** Wenjing Xu, Meng Yang, Xuanlong Du, Hao Peng, Yue Yang, Jitao Wang, Yewei Zhang

**Affiliations:** 1 School of Medicine, Southeast University, Nanjing 210009, China; 2 Department of Ultrasound, State Key Laboratory of Complex Severe and Rare Diseases, Peking Union Medical College Hospital, Chinese Academy of Medical Sciences, Beijing 100005, China; 3 Hepatopancreatobiliary Center, The Second Affiliated Hospital of Nanjing Medical University, Nanjing 210011, China

**Keywords:** multifunctional nanoplatform, sunitinib, photodynamic therapy, photothermal therapy, hepatocellular carcinoma

## Abstract

Hepatocellular carcinoma (HCC) is a tumor that poses a serious threat to human health, with an extremely low five-year survival rate due to its difficulty in early diagnosis and insensitivity to radiotherapy and chemotherapy. To improve the therapeutic efficiency of HCC, we developed a novel multifunctional nanoplatform (SCF NPs) with an amphiphilic polymer (Ce6-PEG2000-FA) and a multitarget tyrosine kinase inhibitor sunitinib. SCF NPs showed superior therapeutical efficiency for HCC due to the synergetic effect of molecular targeted therapy and phototherapy. The Ce6-PEG2000-FA not only serves as a nanocarrier with excellent biocompatibility but also can act as a therapeutic reagent for photothermal therapy (PTT) and photodynamic therapy (PDT). Furthermore, the folic acid group of Ce6-PEG2000-FA enhanced the active targeting performance of SCF NPs. As a multitargeted tyrosine kinase inhibitor, sunitinib in SCF NPs can play a role in molecular targeted therapies, including tumor growth inhibition and anti-angiogenesis. In vivo experiments, SCF NPs showed multimode imaging capabilities, which can be used for tumorous diagnosis and intraoperative navigation. Meanwhile, SCF NPs showed outstanding synergetic tumor inhibition ability. Tumors of SCF NPs group with laser radiation were eradicated without any recrudescence after 14 days of treatment. Such theranostic nanoparticles offer a novel therapeutic tactic for HCC.

## 1. Introduction

Hepatocellular carcinoma (HCC), accounting for 90% of primary liver cancers, is the sixth most common malignancy worldwide and the second leading cause of cancer deaths in China [1,2]. It is mainly caused by chronic hepatitis B and C virus infection [3]. Currently, the potential therapeutic options for early-stage patients with HCC include liver transplantation, surgical resection, and local ablation [4]. However, only 30% of patients present with resectable tumors, and the poor liver reserve capacities and high postoperative recurrence hinder tolerance to surgical treatment [5,6]. HCC is inherently resistant to radiotherapy and chemotherapy due to the expression of multiple drug-resistance genes [7]. Furthermore, HCC is often asymptomatic in the early stage, missing the optimal period for treatment [8]. Therefore, novel diagnostic and therapeutic approaches are essential to be explored for treating this lethal disease.

Sunitinib, a novel small-molecule multitargeted tyrosine kinase inhibitor, has been reported to inhibit HCC growth [9,10,11]. In a recent study, synergizing sunitinib and radiofrequency ablation as a novel therapeutic strategy significantly suppressed HCC growth by triggering the antitumor immune response. Sunitinib, rather than sorafenib (classical first-line drug for HCC), abolished tumor-induced profound immunotolerance to ignite immunological antitumor immune response by significantly suppressing Treg production and PD-1 expression [12]. Additionally, compared to sorafenib, sunitinib has a partial overlap but a significantly distinct target inhibition spectrum [9]. Chemoimmunotherapy of sunitinib combined with anti-PDL-1 antibody has been reported to increase antitumor immunity, inhibit tumor growth, and achieve optimal tumor control [13]. Sunitinib can not only inhibit tumor growth by blocking the signaling pathways of receptor tyrosine kinase, including c-KIT, FLT-3, RET, etc., but also inhibit angiogenesis by targeting vascular endothelial growth factor receptors (VEGFR) and platelet-derived growth factor receptors (PDGFR) [14]. Despite its efficacy, the severe side effects of sunitinib, such as hypertension, myelosuppression, hypothyroidism, and neutropenia, limit its further clinical application in the treatment of HCC [15,16]. Furthermore, the emergence of drug resistance to small-molecule targeted drugs is also a major challenge that cannot be overlooked in the treatment of HCC [17,18]. Among them, the issue of sunitinib resistance has attracted increasingly widespread attention [19,20,21]. Therefore, it is urgent to explore novel therapeutic modalities to tackle the problems of serious side effects and drug resistance of Sunitinib. Compared to traditional therapeutic methods of tumors, phototherapy, including photodynamic therapy (PDT) and photothermal therapy (PTT), has great potential applications in oncotherapy due to its high efficiency, low side effects, and no resistance [22,23,24,25,26]. PDT can transform oxygen into toxic reactive oxygen species (ROS), which have the capability to damage lipids, proteins, and DNA, and further kill cancer cells [27,28,29]. PTT is a promising strategy to rapidly convert localized light into heat in order to kill cancer cells when the temperature exceeds 45 °C [30,31]. However, insufficient oxygen supply (hypoxia) and short half-life (<40 ns) of ROS significantly reduced antitumor efficacy of PDT [32]. The upregulation of the heat shock protein expression and the damage to surrounding tissue severely restrict the clinical application of PTT [24]. Therefore, it is suggested that other therapeutic approaches should be combined with PDT/PTT to address the limitations and enhance the treatment efficacy. In recent years, the application of polymers in the field of medicine and health care has attracted extensive attention. For example, crosslinked polymers with high biocompatibility have attracted much attention in the field of wearable electronics and microfluidic chips for enhancing healthcare status [33,34,35]. Biodegradable polymers are commonly used for controlled-release drug delivery, tissue engineering, and temporary prosthetic implants.

**Scheme 1:**
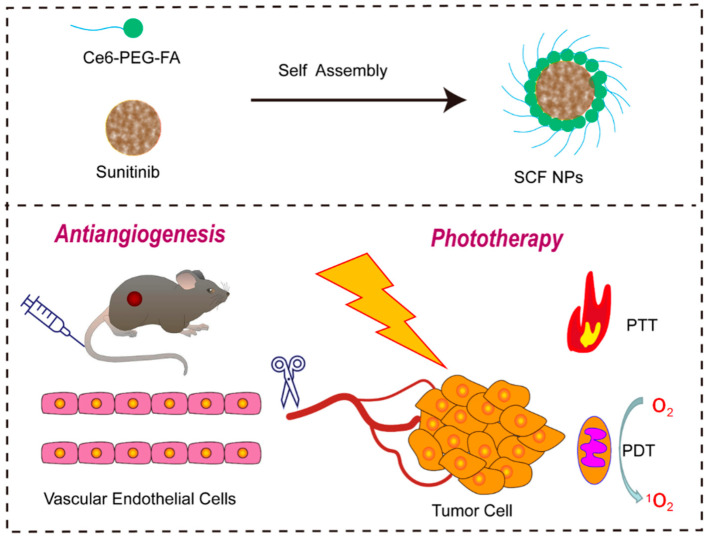
A schematic illustration depicting the fabrication and application of SCF NPs for antivascular and PDT/PTT therapy at the tumor site is presented.

Herein, we employed an amphiphilic polymer (Ce6-PEG2000-FA) to fabricate a novel nanoparticle by self-assembly method, sunitinib@Ce6-PEG2000-FA NPs (SCF NPs), with synergistic antivascular activity and PDT/PTT [36,37]. Sunitinib can induce apoptosis of vascular endothelial cells, thereby inhibiting tumor angiogenesis. Meanwhile, Ce6-PEG2000-FA can achieve PDT and PTT, killing HCC cells. Furthermore, folate receptors on the surfaces of HCC cells can be targeted by the FA molecules of the Ce6-PEG2000-FA to enhance the active targeting capability of NPs [38]. Accordingly, the resulting SCF NPs not only possess excellent passive (enhanced permeability and retention effect) and proactive (folate receptors) targeting ability towards tumors but also present superior synergistic effects with anti-angiogenic therapy and phototherapy (Scheme 1).

## 2. Materials and Methods

### 2.1. Preparation of SCF NPs

The SCF NPs were prepared by the self-assembly method. Firstly, 2 mg of sunitinib and 10 mg of Ce6-PEG2000-FA were dissolved in 2 mL of THF. Subsequently, the mixed solution was added to 10 mL of ultrapure water under sonication. Then, the THF was removed by rapid stirring for 48 h. Finally, the solution filtered through a 220 nm filter was freeze-dried, and the resulting solid powder was stored in the refrigerator at 4 °C.

Remarks: Poly(ethyleneglycol) (PEG), chlorin e6 (Ce6), nanoparticles (NPs), folic acid (FA), tetrahydrofuran (THF). 

### 2.2. Singlet Oxygen Detection 

The ^1^O_2_ generation was detected by a singlet oxygen sensor green (SOSG) reagent. Briefly, 5 μM SOSG was dissolved in SCF NPs aqueous solution. The mixed solution was irradiated by a 660 nm laser (150 mW cm^−2^) for 100 s. The fluorescence was collected every 10 s (excitation wavelength: 404 nm).

### 2.3. Photothermal Effect

Various concentrations of SCF NPs were irradiated by a 660 nm laser (800 mW cm^−2^, 10 min). Furthermore, 100 µg/mL SCF NPs was irradiated with different power of 660 nm laser irradiation. Temperature variations were measured by using a FLIR infrared camera.

### 2.4. Cell Lines and Culture Conditions

Hep-3B cell lines were purchased by the Institute of Biochemistry and Cell Biology, SIBS, CAS (Shanghai, China). Hep-3B Cells were cultured in Dulbecco’s modified Eagle’s medium (DMEM) with 10% fetal bovine serum at 37 °C atmospheres containing 5% CO_2_.

### 2.5. In Vitro Cytotoxicity Assay

The cytotoxicity of Ce6-PEG2000-FA and SCF NPs was estimated by cell viability assay using the Cell Counting Kit-8 (CCK-8) method. Hep-3B and LO2 cells were incubated in 96-well plates in the presence of SCF NPs for 12 h. The light group was illuminated with a 660 nm laser (600 mW cm^−2^) for 3 min, followed by incubation for 12 h. Subsequently, CCK-8 solution was added per well, and the cells were incubated for 2 h at 37 °C in the presence of 5% CO_2_. Finally, the experimental results were detected with a microplate reader. 

### 2.6. Cellular Uptake 

Hep-3B cells were cultured in confocal dishes with incubation for 12 h. Then, 7.6 μg/mL SCF NPs were added into confocal dishes and incubated for 12 h. The cells were washed three times with PBS and fixed with paraformaldehyde for 15 min. DAPI was used to stain the nucleus. The cellular uptake was observed by confocal fluorescence microscope (Olympus FV3000, Tokyo, Japan).

### 2.7. Hemolysis Assay

Fresh blood from nude mice was incubated with saline containing heparin. The whole blood was washed with saline 3 times to obtain a 4% red blood cell (RBC) suspension. Then, 1.0 mL SCF NPs (10, 25, 50, and 100 µg/mL) and the RBC solution (0.2 mL) were mixed and incubated for 6 h. The mixed solution was photographed after centrifugation (3000 rpm/min). Finally, the absorption of supernatant was recorded at 540 nm by ultraviolet spectrophotometer. Hemolysis rate % = (A_SCF NPs_ − A_PBS_)/(A_ultrapure water_ − A_PBS_) × 100%. 

### 2.8. In Vivo Fluorescence Imaging

200 μL of SCF NPs (100 µg/mL) were intravenously administered to the Hep-3B tumor-bearing nude mice. Photos were photographed at 2, 4, 6, 8, and 12 h by the IVIS Lumina K Series III system (Perkin Elmer, Waltham, MA, USA). 

### 2.9. In Vivo Tumor Therapy

Hep-3B cells were subcutaneously injected into the subcutaneous tissue of male nude mice. When the tumor volumes reached about 75 mm^3^, the tumor-bearing nude mice were randomly divided into three groups (control, SCF NPs without laser irradiation, SCF NPs with laser irradiation). After 6 h of tail vein injection of SCF NPs, the tumors of the light group were illuminated by a 660 nm laser for 10 min (600 mW cm^−2^). All groups were treated every 2 days, and the volumes of the tumors were recorded. After two treatments, one nude mouse was taken from each group for hematoxylin and eosin (H&E) staining of tumor tissue. After treatment for 14 days, the nude mice were sacrificed, and the main organs (heart, liver, spleen, lung, and kidney) were dissected. Histopathological examination of main organs was conducted by H&E staining.

## 3. Results and Discussion

### 3.1. Characterization of SCF NPs

**Figure 1:**
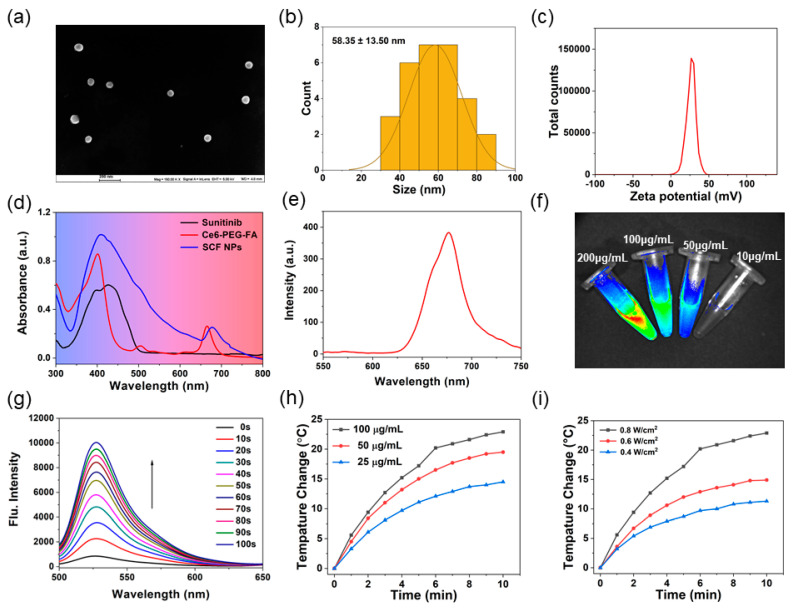
(**a**) SEM image of SCF NPs, scale bar: 200 nm. (**b**) Size distribution of SCF NPs. (**c**) Zeta potential of SCF NPs. (**d**) Ultraviolet absorption spectrum of sunitinib, Ce6-PEG2000-FA, and SCF NPs (Ce6-PEG-FA donated as Ce6-PEG2000-FA). (**e**) The fluorescence emission spectrum of SCF NPs in PBS. (**f**) The fluorescence imaging intensity of various concentration SCF NPs. (**g**) Fluorescence spectra of SOSG mixed with SCF NPs excited by 660 nm laser irradiation. (**h**) Photothermal property of SCF NPs at different concentrations under laser irradiation (660 nm, 800 mW cm^−2^). (**i**) Photothermal property of SCF NPs (100 µg/mL) with different power densities.

As shown in Scheme 1, the water-soluble nanoplatform (named SCF NPs) was obtained by a facile self-assembled approach using amphiphilic polymers (Ce6-PEG2000-FA) (Figure S1) to encapsulate multitarget tyrosine kinase inhibitor (sunitinib) (Figure S2). The detailed synthetic route of SCF NPs was described in the experimental section. The scanning electron microscope (SEM) and transmission electron microscope (TEM) were utilized to evaluate the morphologies of SCF NPs (Figure 1a, Figures S3 and S4), and the results recapitulated that the prepared nanoparticle was uniformly spherical morphology with a diameter of around 58 nm (Figure 1b) and the Zeta potential (Figure 1c) was +25.93 mV, suggesting successful synthesis of the nanoparticles. The suitable size of SCF NPs can facilitate nanoparticles targeting tumor tissue through enhanced permeability and retention (EPR) effect. After incubation with PBS for 14 days, no obvious aggregation or precipitate was observed, indicating superior solubility and good stability of SCF NPs. (Figure S5). In Figure 1d, SCF NPs displayed two obvious absorption peaks at 411 nm and 678 nm, a slight red shift could be observed compared to free Ce6-PEG2000-FA, which proved the successful loading of drugs (Figure 1d). Furthermore, the clear fluorescence peak (Figure 1e) and fluorescence photos of various concentrations (10, 50, 100, 200 µg/mL) (Figure 1f) indicated the potential of SCF NPs for fluorescence imaging applications. To assess the efficiency of PDT in vitro, a typical molecular singlet oxygen sensor green (SOSG) was used as the probe to detect the ^1^O_2_ generation in SCF NPs aqueous solution via detecting the change of fluorescence intensity spectrum. Under laser irradiation (150 mW cm^−2^), the fluorescence intensity increased gradually, which proved that SCF NPs possess remarkable ^1^O_2_ generation performance (Figure 1g). Furthermore, the ^1^O_2_ generation ability of SCF NPs was further verified by monitoring the changes in the ultraviolet absorption spectrum of 1,3-diphenylisobenzofuran (DPBF) at 418 nm with 660 nm laser irradiation.

The absorption intensities of DPBF mixed with SCF NPs in PBS (pH 7.4) time-dependently decrease under the 660 nm laser irradiation (Figure S6). Meanwhile, after the conjugation of PEG2000 with Ce6, SCF NPs still exhibited superior ^1^O_2_ photogeneration owing to the action of Ce6-PEG2000-FA (Figure S7). These results unambiguously demonstrated that SCF NPs can be employed as an outstanding PDT therapeutic agent for tumor therapy. Considering the existing near-infrared-region (NIR) absorption of SCF NPs, the photothermal effect was further investigated in PBS aqueous solution. The temperature changes of SCF NPs at different concentrations were investigated using a thermal imaging device under illumination conditions (800 mW m^−2^). The temperature changes of SCF NPs increased gradually with the increasing concentrations (Figure 1h). The temperature of SCF NPs increased by 14.5 °C even at a low concentration (25 µg/mL), and the photothermal performance may be attributed to the presence of Ce6. The photothermal properties of Ce6 were retained after successful conjugation with PEG2000, and with increasing laser power, a corresponding increase in temperature was observed at a concentration of 100 µg/mL of SCF NPs (Figure 1i). Taken together, these results suggested that SCF NPs could serve as a potential nanoplatform for both PDT and PTT. 

### 3.2. Cellular Experiment of SCF NPs

**Figure 2:**
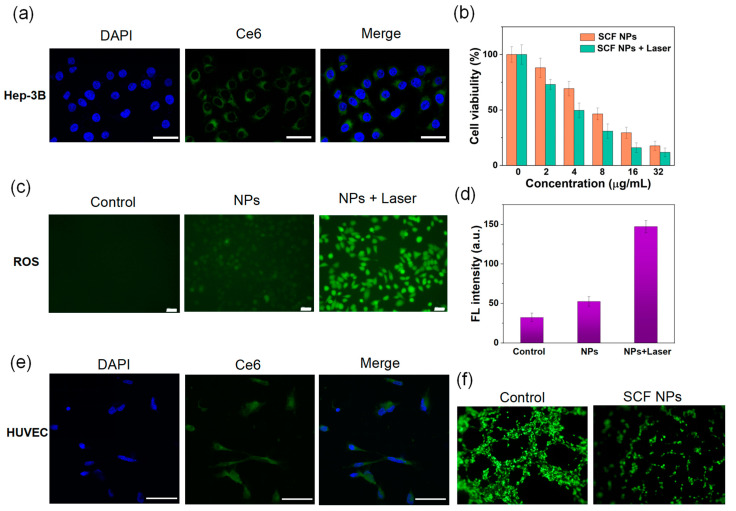
(**a**) Cellular uptake of SCF NPs in Hep-3B cells, scale bar: 30 µm. (**b**) In vitro cytotoxicity of SCF NPs for Hep-3B cell measured by CCK-8. (**c**) DCFH-DA probe staining to detect the generation of ^1^O_2_. Green fluorescence represents the generation of ^1^O_2_. Scale bar: 50 µm. (**d**) Fluorescence intensity quantitative analysis of DCFH-DA. (**e**) Cellular uptake of SCF NPs in HUVEC cells, scale bar: 30 µm. (**f**) In vitro anti-angiogenesis assay of control group and SCF NPs group.

The intracellular uptake of SCF NPs was observed using a confocal fluorescence microscope with Ce6 as a fluorescent probe. As shown in Figure 2a, the green fluorescence of Ce6 could be observed within the cytoplasm of Hep-3B cells by confocal fluorescence microscopy, suggesting that SCF NPs are efficiently endocytosed by tumor cells. Biosafety assessment is critical before nanocarriers are used for biomedical research. The Hep-3B and human normal hepatocytes (LO2) were employed to evaluate the biocompatibility of Ce6-PEG2000-FA. CCK-8 assays were conducted to detect the cytotoxicity of Ce6-PEG2000-FA. As described in Figure S8, the result of CCK-8 indicated that the Ce6-PEG2000-FA showed superior biosafety. Both cell lines maintained high cell viability (>90%) even at concentrations greater than 200 µg/mL. As the nanocarrier of SCF NPs, Ce6-PEG2000-FA not only have a good encapsulation effect but also show little toxicity to normal cell and tumor cell. Then, the efficacy of combination therapy comprising phototherapy and molecular targeted therapy was evaluated. As indicated in Figure 2b, the cell survival rates of tumor cells treated with SCF NPs with or without laser radiation were concentration dependent. It is worth noting that dark toxicity of SCF NPs is rather high, with 46.5% cell viability at a concentration of 8 µg/mL. This phenomenon may be attributed to the successful loading of sunitinib, effective cellular uptake of nanoparticles, and smooth intracytoplasmic release of sunitinib. Importantly, SCF NPs exhibited strong phototoxicity, with 30.9% cell viability at a concentration of 8 µg/mL. The IC50 values of SCF NPs with or without laser irradiation are 7.8 and 4.3 µg/mL for Hep-3B cells, respectively. Therefore, SCF NPs showed remarkable synergistic therapeutic outcomes based on phototherapy and molecular targeted therapy. Subsequently, we further investigated the intracellular ROS generation of various groups by using DCFH-DA as the fluorescence probe. In Figure 2c, the strongest fluorescence emission could be detected in the group of MCS NPs with laser irradiation. The quantitative analysis of the fluorescence intensity further confirmed this result (Figure 2d). Notably, the group of SCF NPs without light irradiation also displayed a slight fluorescent signal, which may be attributed to sunitinib-induced generation of reactive oxygen species (ROS) [39,40]. In order to verify the antivascular properties of SCF NPs, a blood vessel formation experiment was performed. As displayed in Figure 2e,f, SCF NPs can be successfully taken up by HUVEC cells, and SCF NPs group could effectively fight against angiopoiesis (vascular length, tightness, and intersections significantly decreased) than the control group. Therefore, a synergistic therapeutic strategy combining phototherapy with molecular targeted therapy can effectively eliminate tumor cells in vitro. 

### 3.3. Multimodal Imaging and In Vivo Experiments

**Figure 3:**
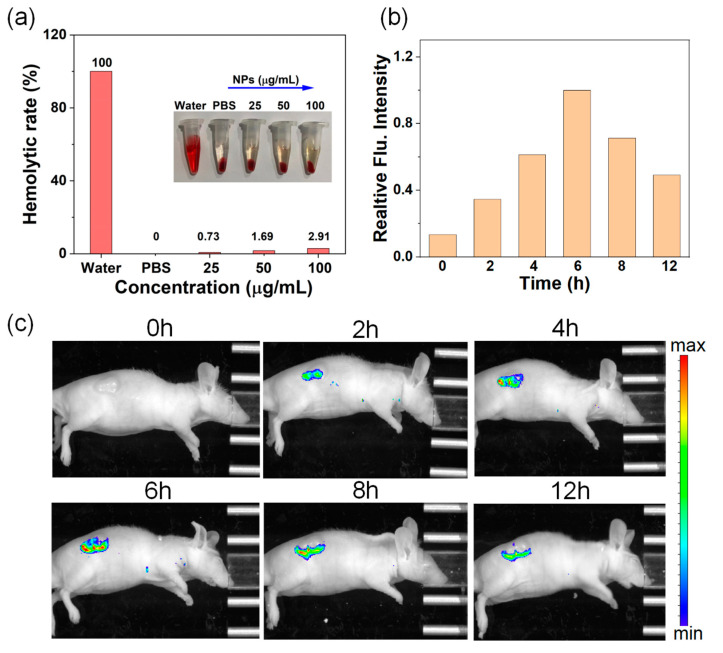
(**a**) Hemolysis experiment with different concentrations (25, 50, 100 µg/mL) of SCF NPs. (**b**) Quantification of the fluorescence signal of Hep-3B tumor-bearing mice before and after intravenous injection with SCF NPs. (**c**) Real-time fluorescence imaging of SCF NPs.

**Figure 4:**
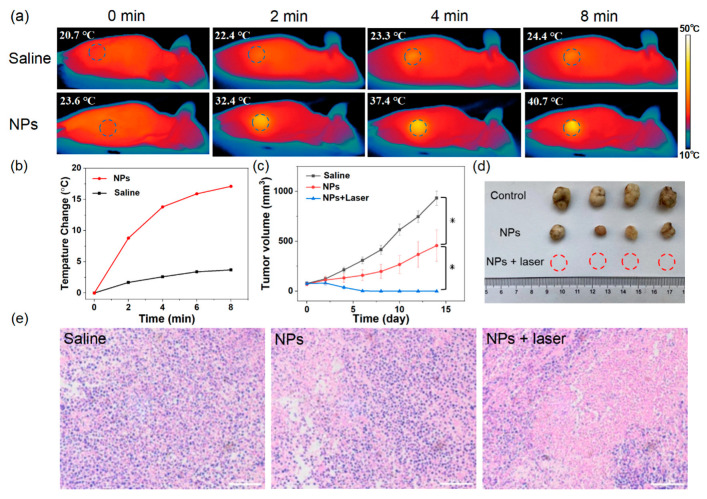
(**a**) IR thermal images of mice bearing Hep-3B tumors injected with saline or SCF NPs, irradiated with 660 nm laser (600 mW cm^−2^). (**b**) The curves of temperature changes at the tumor site under laser irradiation. (**c**) Tumor volume growth curves of various groups. * *p* < 0.05. (**d**) Images of dissected tumors from different groups (Control, SCF NPs, SCF NPs with laser radiation). (**e**) H&E staining of tumor tissue for each group, scale bars: 50 µm.

**Figure 5:**
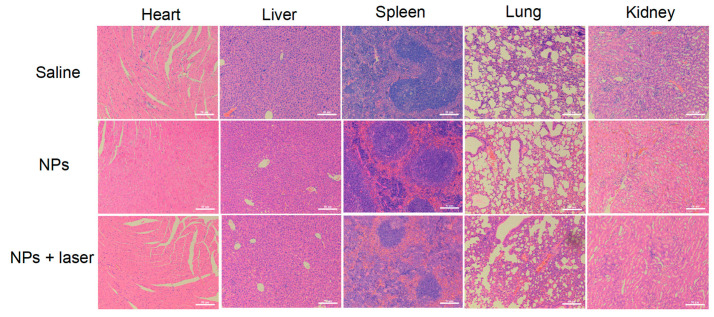
H&E staining for major organ sections (heart, liver, spleen, lung, and kidney) from different groups of mice. Scale bar: 50 μm.

The imaging performance of nanoparticles is of great significance for early diagnosis, targeted treatment, and intraoperative guidance of tumors. To investigate the in vitro imaging properties of SCF NPs, a hemolysis experiment was performed to assess the biocompatibility of nanoparticles. As illustrated in Figure 3a, the hemolysis rate (2.91%) was less than 5% even at a concentration of 100 µg/mL SCF NPs, demonstrating satisfactory biosafety in vivo. Subsequently, we used real-time fluorescence imaging to assess the targeting performance of SCF NPs in vivo. Figure 3b,c showed the real-time fluorescence imaging and fluorescence intensity change in vitro. Before intravenous injection of SCF NPs, no fluorescence signals at the tumor site could be observed. After intravenous injection of nanoparticles, a gradual increase in fluorescence signal at the tumor site was observed, reaching a maximum after approximately six hours, followed by a gradual decrease. The results of fluorescence imaging experiments confirmed the superior tumor-targeting properties of SCF NPs, which may be attributed to the size-dependent passive targeting properties and the folate-relevant active targeting properties. Owing to the outstanding photothermal peculiarity of SCF NPs, after 6 h intravenous injection of SCF NPs, the real-time photothermal photos at the tumor site can be visually evaluated by the infrared camera. The thermal imaging photos indicated the effective enrichment of SCF NPs at tumor sites. As shown in Figure 4a,b, the temperature of the tumor area in mice injected with SCF NPs can be elevated by more than 17 °C upon 660 nm laser irradiation, providing an effective photothermal treatment. In comparison, the temperature of mice injected with saline only increased slightly by about 3.7 °C. In a word, the multimodal imaging of SCF NPs could provide effective diagnosis and precise intraoperative navigation. The excellent tumor-targeting ability and imaging performance of SCF NPs encourage us to further evaluate therapeutic efficacy in vivo. To validate the antitumor effect of SCF NPs in vivo, tumor volumes were recorded every two days during 14 days of treatment. As shown in Figure 4c,d, compared to the control group, the tumor growth was effectively inhibited but not eliminated entirely for the group treated with SCF NPs. Noteworthy, we surprisingly observed that the SCF NPs group with laser radiation can completely eradicate tumors after two or three treatments without any recrudescence. The results indicate that SCF NPs can remarkably inhibit tumor growth by combining phototherapy and molecular targeted therapy. After two treatments, tumors were removed from nude mice and stained for hematoxylin and eosin (H&E). The histopathology results indicate that SCF NPs with laser radiation could effectively induce cell apoptosis and necrosis of tumor tissues (Figure 4e). To evaluate the toxicology of SCF NPs on major organs (heart, liver, spleen, lung, and kidney), after 14 days of treatment, mice were sacrificed and dissected to recover major organs, which were subsequently fixed in 10% buffered formalin and embedded in paraffin wax for routine histological examination via H&E staining (Figure 5). The experimental results showed no obvious damage, which indicating the prepared SCF NPs have no obvious toxicity. These results indicated that SCF NPs exhibited promising antitumor efficacy through PDT/PTT and molecular targeting therapy.

## 4. Conclusions

In summary, we developed a multifunctional nanoplatform (SCF NPs) by utilizing a novel amphiphilic polymer (Ce6-PEG2000-FA) to deliver a multitarget tyrosine kinase inhibitor (sunitinib). The Ce6-PEG2000-FA exhibited outstanding biocompatibility, and the final prepared nanoparticles possess a diameter of around 58 nm, which can target tumor tissue through the EPR effect. The folic acid group in Ce6-PEG2000-FA can enhance the active targeting performance of nanoparticles. The SCF NPs showed antivascular ability due to the successful loading of sunitinib. The SCF NPs demonstrated superior therapeutic effects based on PDT, PTT, and molecular targeted therapy. Simultaneously, SCF NPs exhibited remarkable photothermal and fluorescence imaging properties, which can be utilized for tumor diagnosis and intraoperative navigation. This novel sunitinib-based nanoplatform for multimodal therapy offers a promising tactic for the treatment of HCC.

## Data Availability

The data that support the findings of this study are available from the corresponding author upon reasonable request.

## Supplementary Materials

The following supporting information can be downloaded at: https://www.mdpi.com/article/10.3390/mi14030613/s1, Figure S1: The chemical formula of Ce6-PEG2000-FA; Figure S2: The chemical formula of sunitinib; Figure S3: SEM images of SCF NPs; Figure S4: TEM image of SCF NPs; Figure S5: The photo of PBS aqueous solution after incubuating SCF NPs for 14 days; Figure S6: The degradation of DPBF with the presence of SCF NPs under 660 nm laser irradiation; Figure S7: The degradation of DPBF with the presence of Ce6-PEG2000-FA under 660 nm laser irradiation; Figure S8: Cytotoxicity assay of Hep-3B and LO2 cell incubuated with different concentrations (20, 40, 60, 80, 100, 200 µg/mL) of Ce6-PEG2000-FA.
