# Supplementary material for: Multifunctional Nanoplatform Based on Sunitinib for Synergistic Phototherapy and Molecular Targeted Therapy of Hepatocellular Carcinoma
Source: Micromachines (Basel). 2023 Mar 7;14(3):613. doi: 10.3390/mi14030613 (PMC10059596; doi:10.3390/mi14030613)
Supplement: Supplementary file 1 [file micromachines-14-00613-s001.zip › micromachines-2195718-supplementary.pdf]

Supporting information

Multifunctional Nanoplatform Based on Sunitinib for Synergistic Phototherapy and Molecular Targeted Therapy of Hepatocellular Carcinoma

Wenjing Xu[1,†], Meng Yang[2,†], Du Xuanlong[1], Hao Peng[1], Yue Yang[1], Jitao Wang[1], Yewei Zhang[3,*]

[1] School of Medicine, Southeast University, Nanjing 210009, China.

[2] Department of Ultrasound, State Key Laboratory of Complex Severe and Rare Diseases, Peking Union Medical College Hospital, Chinese Academy of Medical Sciences and Peking Union Medical College, Beijing, China.

[3] Hepatopancreatobiliary Center, The Second Affiliated Hospital of Nanjing Medical University, Nanjing, 210011, China.

* Correspondence: zhangyewei@njmu.edu.cn

[†] These authors contributed equally.

Figures

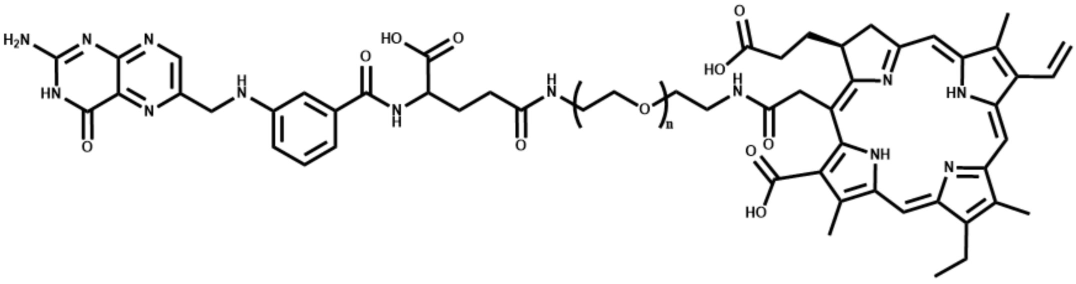

Figure S1. The chemical formula of Ce6-PEG2000-FA.

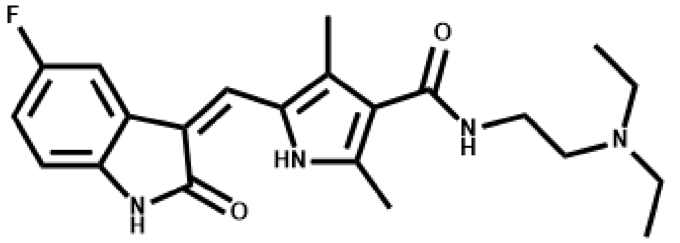

Figure S2. The chemical formula of sunitinib.

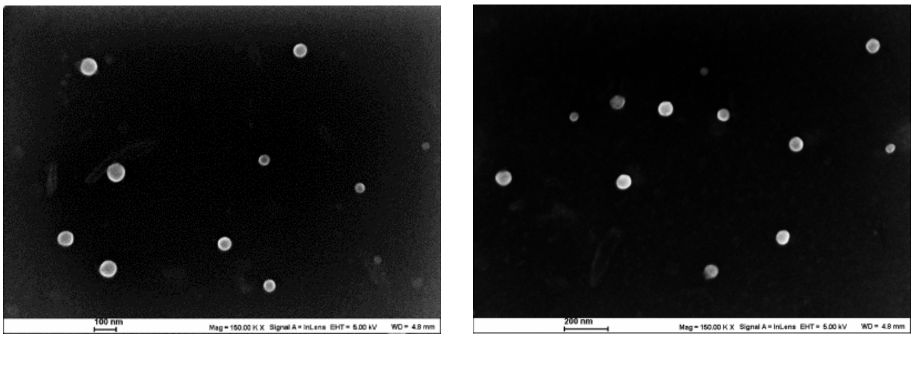

Figure S3. SEM images of SCF NPs.

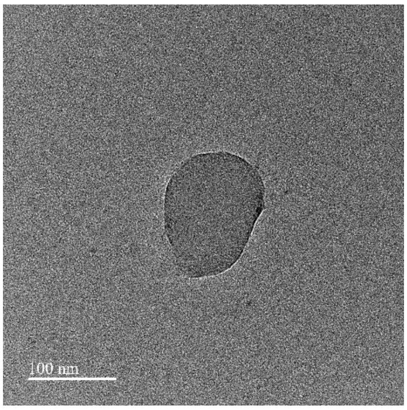

Figure S4. TEM image of SCF NPs.

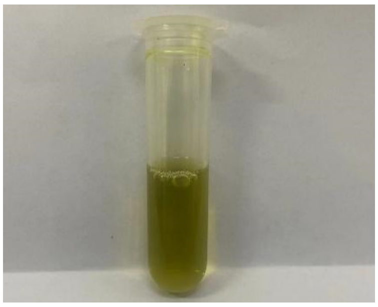

Figure S5. The photo of PBS aqueous solution after incubuating SCF NPs for 14 days.

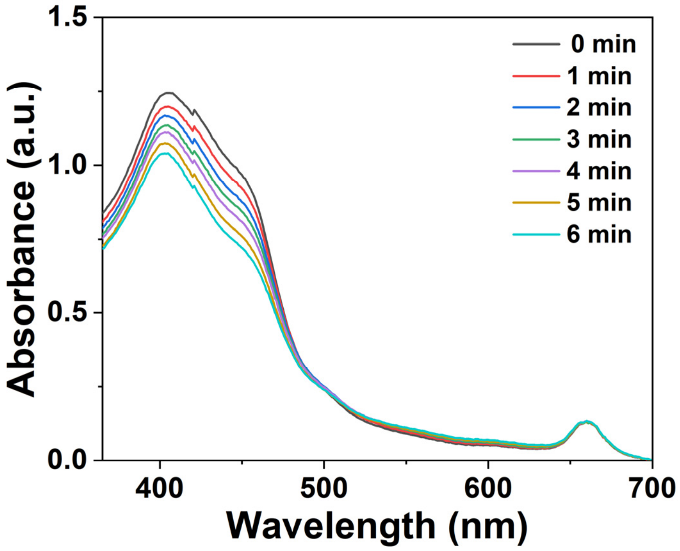

Figure S6. The degradation of DPBF with the presence of SCF NPs under 660 nm laser irradiation.

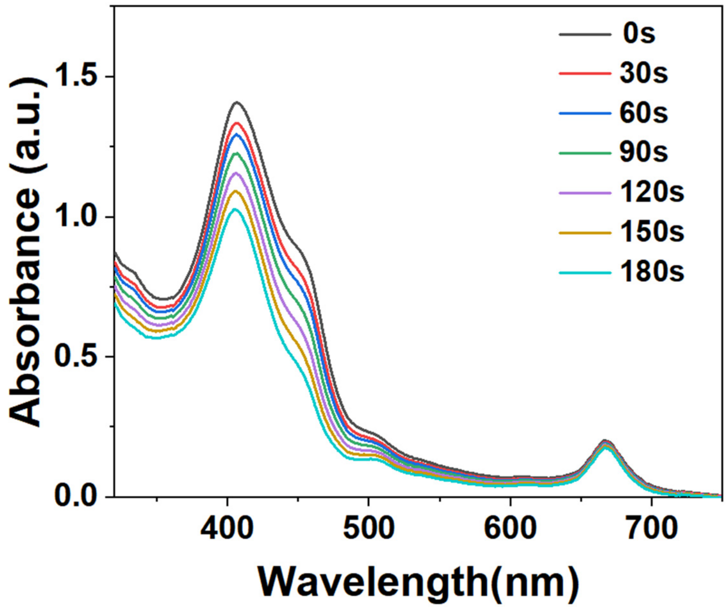

Figure S7. The degradation of DPBF with the presence of Ce6-PEG2000-FA under 660 nm laser irradiation.

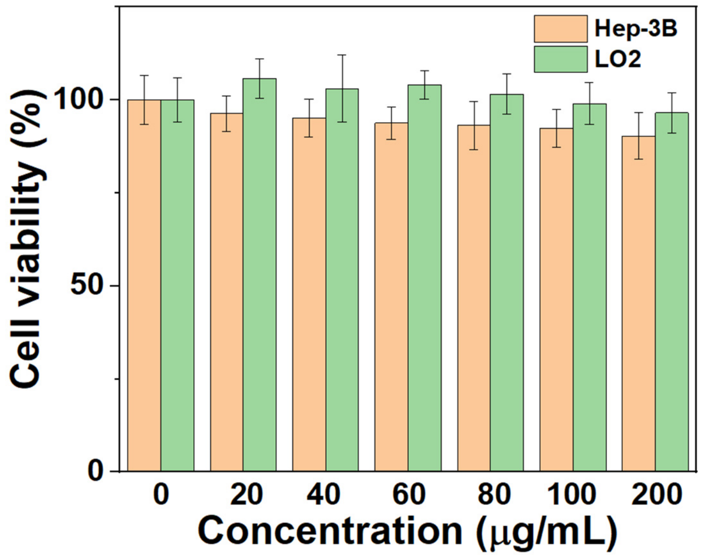

Figure S8. Cytotoxicity assay of Hep-3B and LO2 cell incubuated with different

concentrations (20, 40, 60, 80, 100, 200 μg/mL) of Ce6-PEG2000-FA.
